# The effectiveness of Tai Chi for postpartum depression: A protocol for systematic review and meta-analysis

**DOI:** 10.1097/MD.0000000000028176

**Published:** 2021-12-10

**Authors:** Haoyu Tian, Shengnan Han, Jing Hu, Xiangyu Peng, Wei Zhang, Wanyu Wang, Xianghua Qi, Jing Teng

**Affiliations:** a Shandong University of Traditional Chinese Medicine, China; b Affiliated Hospital of Shandong University of Traditional Chinese Medicine, China.

**Keywords:** meta-analysis, postpartum depression, protocol, systematic review, Tai Chi

## Abstract

**Background:**

As a specific type of depression, postpartum depression (PPD) causes an adverse hazard to the mother's physical and mental health. Considering the safety requirements for lactation and the expectation of the rapid response to treatment, the search for safe and effective alternative therapies has attracted wide attention. Tai Chi, a traditional Chinese exercise therapy, has been widely used to relieve the symptoms and complications of patients with PPD, which the clinical efficacy is questioned. We conduct a comprehensive systematic review and meta-analysis to find clinical medical evidence of Tai Chi in the treatment of PPD.

**Methods:**

PubMed, Embase, Cochrane Library, Web of Science, China National Knowledge Infrastructure, China Science, and Technology Journal Database and Chinese Biomedical Literature Database will be searched from their inception of databases to September 30, 2021. Two reviewers will select articles, extract data, and assess the risk of bias independently. Any disagreement will be resolved by discussion with the third reviewer. Review Manager 5.3 software will be used for data synthesis. The Cochrane risk of bias assessment tool will be used to assess the risk of bias.

**Results:**

This study will conduct a comprehensive literature search and provide a systematic synthesis of current published data to explore the effectiveness of Tai Chi for PPD.

**Conclusions:**

The findings of our study will provide updated evidence to determine whether Tai Chi is an effective intervention for patients with PPD, which will help clinicians make a better alternative treatment schedule of PPD patients and provide a reliable basis for health-related policymakers.

**Study registration number:**

CRD42021276676.

## 1 Introduction

As a specific type of depression, postpartum depression (PPD), which causes a second psychological trauma to women who have just experienced the pain of childbirth and have not fully recovered, leads to worse medical harm than general depression. PPD causes an adverse hazard to the mother's physical and mental health, affects the baby's future development and growth, and generates irreparable family burdens and socio-economic problems.^[1,2]^ Considering the safety requirements for lactation and the expectation of the rapid response to treatment, most women with PPD prefer psychological to pharmacological treatments, and a desire to avoid medication,^[3]^ which the search for safe and effective alternative therapies has attracted wide attention. The prevalence of PPD varies from 0.5% to 60.8% around the world and from 3.5% to 63.3% in Asian countries, as measured using the Edinburgh Postpartum Depression Scale (EPDS).^[4]^ Due to the rapid changes in postpartum hormone levels, it not only trigger psychological symptoms such as depressed mood, loss of interest, anhedonia, feelings of guilt or worthlessness, impaired concentration, suicidal thoughts, but also lead to somatic symptoms such as galactostasis, fatigue, insomnia, nausea and vomiting, difficulty in caring for young children, sleep and appetite disturbance, etc.^[5,6]^ PPD is a representative psychosomatic disease, which psychological symptoms and somatic symptoms could interact with each other. Somatic symptoms have a significant impact on symptomology and treatment outcomes of depression.^[7]^ Moreover, depression may have a close relationship with the vast majority of somatic diseases.^[8]^ To our regret, clinical treatments emphasize the adjustment of its psychological state, while ignoring the joint regulation of somatization and prevention of more serious physical diseases.

Tai Chi originated in ancient China for thousands of years, which was formed through historical precipitation under the guidance of the essence of Chinese folk and military martial arts, breathing and meditative techniques, and traditional Chinese medicine theory.^[9,10]^ Just like the academic thoughts of traditional Chinese medicine such as “Use exercise to regulate emotions”, “the body and the spirit are jointly regulated”, it is also a kind of exercise therapy that harmonizes yin-yang and promotes homeostasis between body and mind.^[11]^ It is widely loved by the Chinese people and even the Asian people. Tai Chi training can be launched in a group-based pattern which may impel practitioners to stay motivated and enthusiastic to continue practicing.^[12]^ This is especially because of the social benefits yielded by the communications and interactions regarding Tai Chi.^[13]^ In previous studies, Tai Chi can alleviate or treat somatic or psychological diseases, the former such as fibromyalgia,^[14]^ cardiovascular disease,^[15,16]^ osteoarthritis,^[17]^ and chronic obstructive pulmonary disease,^[18]^ the latter such as cognitive impairment,^[19]^ depression and anxiety,^[20]^ and it can also treat some typical psychosomatic disease, such as insomnia in survivors of breast cancer^[21]^ and perimenopausal psychosomatic symptoms,^[22]^ and physical and psychosocial impairment among individuals with impaired physical mobility.^[23]^

The psychological and physiological dual therapeutic effect of Taijiquan coincides with the characteristics of physical and mental comorbidities of PPD. Tai Chi has been widely used as an alternative to recommended treatments for PPD in China, however, its clinical efficacy is questioned. Notably, there is a promising evidence base for exercise-based interventions in preventing and treating depressive symptoms of PPD.^[24,25]^ Up to now, although some researchers have done systematic reviews and meta-analysis on the effect of Tai Chi on depression, there is no relevant research on its special type--PPD. It is necessary to implement a comprehensive systematic review and meta-analysis to evaluate the therapeutic effects of Tai Chi for PPD.

## 2 Methods

### 2.1 Protocol and registration

The study protocol has been registered on international prospective register of systematic review (PROSPERO) in March 2021. The trial registration number of PROSPERO is CRD42021276676.

### 2.2 Ethic approval

Ethical approval will not be necessary since this systematic review and meta-analysis only uses published papers, which will not reveal personal privacy and violate human rights.

### 2.3 Inclusion criteria for study selection

#### 2.3.1 Types of studies

Only randomized controlled trials can be included in our research, case reports, animal experiments, and reviews will be excluded, and there will be no language restrictions in the selection of literature.

#### 2.3.2 Types of patients

Regardless of their nationality, age, race, patients who meet the diagnostic criteria for PPD can be included as participants.

#### 2.3.3 Types of interventions

The treatment method of the experimental group will be Tai Chi training alone or combined with medication and placebo treatment. There will be no restrictions on the genre and contact frequency of Tai Chi. The control group will be treated with medication or placebo, and trials involving other exercise therapies will be excluded.

#### 2.3.4 Types of outcome measures

The diagnosis of PPD will be based on The Diagnostic and Statistical Manual of Mental Disorders (DSM-5), published by the American Psychiatric Association.^[26]^ The primary efficacy measure is EPDS and the second measure is the Hamilton Depression Scale. EPDS is an objective scale that specifically evaluates PPD, and it is widely used to evaluate the treatment efficacy of this disease. Hamilton Depression Scale is the most widely used scale for diagnosis and assessment of depression and it comprises 24 items of depressive symptoms, which are scored in 5 grades.^[27]^

### 2.4 Search strategy

PubMed, Embase, Cochrane Library, Web of Science, China National Knowledge Infrastructure, China Science, and Technology Journal Database and Chinese Biomedical Literature Database will be searched from their inception of databases to September 30, 2021. In order to prevent the omission of related resources in non-electronic versions, we will also search Baidu Scholar, Google Scholar, the International Clinical Trials Registry Platform, and the Chinese Clinical Trial Registry.

### 2.5 Data collection and analysis

#### 2.5.1 Selection of studies

**Figure 1:**
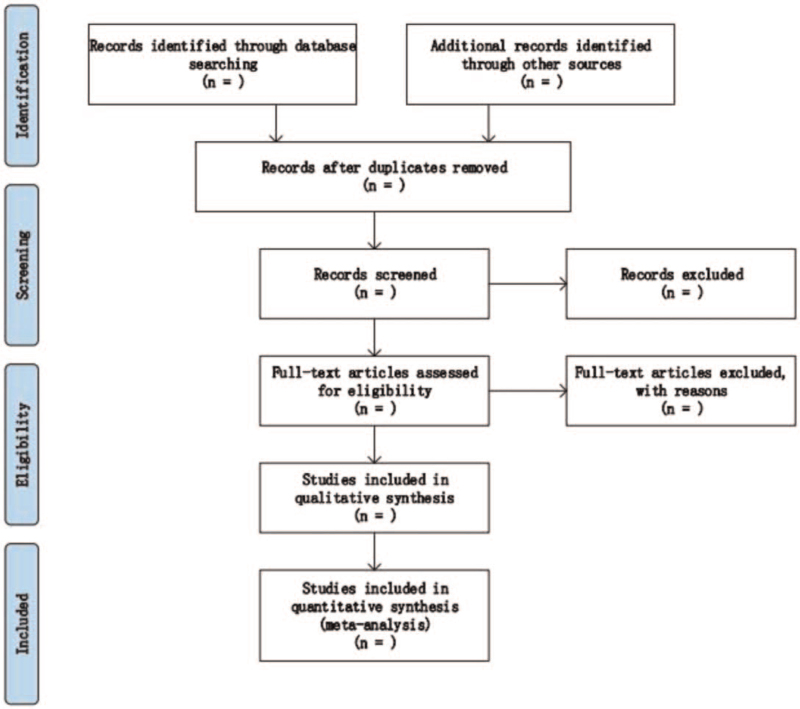
Preferred Reporting Items for Systematic Review and Meta-analysis (PRISMA) flowchart.

Two researchers independently screened and extracted data based on the inclusion criteria. The researcher will discuss and analyze the inconsistency of the 2 sets of results. If there are still differences, the third researcher will arbitrate. For ambiguities in the data, we will contact the author to make improvements. The entire process of research screening, extraction, and inclusion is shown in the Preferred Reporting Items for Systematic Review and Meta-analysis (PRISMA) flowchart (Fig. 1).

#### 2.5.2 Data collection and management

The following key information will be extracted: the first author, publication year, journal, study design, sample size, trial location, age, geographic population, health status, duration and follow-up, frequency, intensity, Tai Chi style, control intervention, baseline and outcome data, and adverse events.

#### 2.5.3 Assessment of risk of bias in included studies

Under the guidance of risk of bias assessment tool of the Cochrane Handbook, 2 researchers will independently conduct 3-level evaluations (“high”, “unclear”, or “low”) on following aspects: random sequence generation; allocation concealment; blinding of participants and personnel; blinding of outcome assessment; incomplete outcome data; selective reporting; other bias. If there are differences in the evaluation, the third researcher will make the final judgment.

#### 2.5.4 Measures of the treatment effect

For the binary variable data, we will use odds ratios and risk ratio as the combined statistics. For quantitative variable data, standardized mean difference is used for the combined effect statistics, and 95% confidence intervals are used for the above 2 types of data.

#### 2.5.5 Dealing with missing data

We will directly contact the authors of the literature by e-mail to clarify the ambiguous key information, or to supplement the missing valid data. If the above methods cannot effectively solve the problem, the literature will be excluded.

#### 2.5.6 Assessment of heterogeneity

The χ^2^ test and the I^2^ value were used to evaluate the heterogeneity between the studies. If *P* > .1 and I^2^ < 50%, it means that the studies have satisfactory homogeneity, and the fixed effect model is used for statistical analysis; on the contrary, under the prerequisite of excluding clinical heterogeneity, the random effect model will be selected.

#### 2.5.7 Assessment of reporting bias

If the number of included studies is not less than 10, we will observe the symmetry of the funnel chart to assess reporting bias. Egger test and Begg test will also be used to assess reporting bias.

#### 2.5.8 Data synthesis

Data analysis will use software Review Manager 5.4.1 provided by Cochrane Collaboration. For the binary variable data, we will use odds ratios and risk ratio as the combined statistics. For quantitative variable data, standardized mean difference is used for the combined effect statistics, and 95% confidence intervals are used for the above 2 types of data. We will use different effect models based on the results of the heterogeneity test, if the results are homogeneous, the fixed effect model will be used, otherwise, the random effect model will be used.

#### 2.5.9 Subgroup analysis

In order to analyze the potential reasons for the high heterogeneity, we will conduct a subgroup analysis based on the actual situation. The patient's age, marriage and childbearing history, educational background, and frequency of Tai Chi training will be the main points of concern.

#### 2.5.10 Sensitivity analysis

We will determine the reliability and reliability of the results by eliminating documents with high risk of bias or high abnormal values. After re-incorporating the literature, if the relevant results undergo reversal changes, we will cautiously explain the conclusions.

## 3 Discussion

This is the first systematic review and meta-analysis to conduct a comprehensive literature search and provide a systematic synthesis of current published data to explore the effectiveness of Tai Chi for PPD. Our protocol for systematic review and meta-analysis will integrate available evidence by rigorous procedure and provide evidence in support or against the hypothesis that Tai Chi could alleviate the symptom of PPD patients, which will help clinicians make a better alternative treatment schedule of PPD patients and provide reliable basis for health-related policymakers.

## Author contributions

**Conceptualization:** Haoyu Tian.

**Data curation:** Shengnan Han, Jing Hu.

**Formal analysis:** Shengnan Han, Jing Hu.

**Methodology:** Jing Teng.

**Project administration:** Xianghua Qi, Jing Teng.

**Supervision:** Jing Teng.

**Validation:** Haoyu Tian, Xianghua Qi, Jing Teng.

**Writing – original draft:** Haoyu Tian, Xiangyu Peng, Wei Zhang, Wanyu Wang.
